# 3D Printed Multi‐Cavity Soft Actuator with Integrated Motion and Sensing Functionalities via Bio‐Inspired Interweaving Foldable Endomysium

**DOI:** 10.1002/advs.202409060

**Published:** 2024-11-26

**Authors:** Zhonggui Fang, Shaowu Tang, Yinyin Su, Xiaohuang Liu, Sicong Liu, Juan Yi, Zheng Wang, Jian S. Dai

**Affiliations:** ^1^ Department of Mechanical and Energy Engineering Southern University of Science and Technology Shenzhen 518000 China; ^2^ Department of Mechanical Engineering The University of Hong Kong Hong Kong 999077 China

**Keywords:** bio‐inspired design, integrated motion and sensing, single‐material foldable multicavity, soft actuator

## Abstract

The human muscle bundle generates versatile movements with synchronous neurosensory, enabling human to undertake complex tasks, which inspires researches into functional integration of motions and sensing in actuators for robots. Although soft actuators have developed diverse motion capabilities utilizing the inherent compliance, the simultaneous‐sensing approaches typically involve adding sensing components or embedding certain‐signal‐field substrates, resulting in structural complexity and discrepant deformations between the actuation parts with high‐dimensional motions and the sensing parts with heterogeneous stiffnesses. Inspired by the muscle‐bundle multifiber mechanism, a multicavity functional integration (McFI) approach is proposed for soft pneumatic actuators to simultaneously realize multidimensional motions and sensing by separating and coordinating active and passive cavities. A bio‐inspired interweaving foldable endomysium (BIFE) is introduced to construct and reinforce the multicavity chamber with optimized purposive foldability, enabling 3D printing single‐material fabrication. Performing elongation, contraction, and bidirectional bending, the McFI actuator senses its spatial position, orientation, and axial force, based on the kinematic and sensing models built on multi‐cavity pressures. Two McFI‐actuator‐driven robots are built: a soft crawling robot with path reconstruction and a narrow‐maneuverable soft gripper with object exteroception, validating the practicality in stand‐alone use of the actuator and the potential for intelligent soft robotic innovation of the McFI approach.

## Graphical abstract

Inspired by the muscle‐bundle multifiber mechanism, the multicavity functional integration (McFI) approach is proposed for soft pneumatic actuators to simultaneously realize multidimensional motions and sensing, by separating and coordinating active and passive cavities. The multicavity structure is constructed and reinforced by the bio‐inspired interweaving foldable endomysium (BIFE). This approach is validated by the trajectory reconstruction crawler and the exteroception gripper.

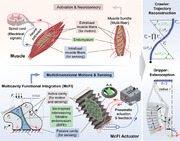

## 1 Introduction

Muscles generate versatile movements with embedded neural feedback enable human to perform high‐dexterous maneuvers, undertake complex tasks, and adapt to unstructured environments.^[^ ^1^, ^2^, ^3^ ^]^ With inherent compliance similar to biological organisms, soft robots exhibit a promising potential to provide biomimetic solutions for applications in unstructured environments.^[^ ^4^, ^5^, ^6^ ^]^ Endowing soft robots with diverse motions and simultaneous sensing is essential to obtain dexterous and intelligent manipulations, which further improves the physical interaction capabilities and broadens application scenarios.

As the crucial component of soft robots, soft pneumatic actuators with a variety of motions have been developed, including elongation,^[^ ^7^ ^]^ contraction,^[^ ^8^, ^9^ ^]^ bending,^[^ ^10^, ^11^ ^]^ twisting,^[^ ^12^, ^13^ ^]^ and multidimensional motions by motion‐coupling.^[^ ^14^, ^15^, ^16^, ^17^, ^18^ ^]^ These movements are usually achieved by structural design with asymmetrically distributed constraints, resulting in the anisotropic stiffness and desired deformation when subjected to driving input. Due to the easy customization of the motion and mechanical property using the creases as the embedded constraints,^[^ ^19^ ^]^ origami‐patterned structures attract increasing interest in constructing soft actuators with desired motion and enhanced stiffness.^[^ ^19^, ^20^, ^21^, ^22^ ^]^

For operations requiring dexterous maneuvers, the multi‐actuator configuration is usually utilized to coordinate motions through the arrangement of actuators, generating multiple degrees of freedom (DOF) manipulation.^[^ ^23^, ^24^, ^25^, ^26^ ^]^ Among pneumatic soft actuators, the airtight cavity is necessary^[^ ^27^, ^28^, ^29^ ^]^ to obtain large continuous deformations and high load‐weight ratio. For soft actuators to perceive motion and external interactions, the integration of sensing function is fundamental and hence provides feedback for closed‐loop control. However, it is challenging to develop a compact soft actuator with integrated sensing, due to the highly coupled large‐strain deformations of the soft material during the multidimensional movements.

There have been various efforts devoted to enable sensing for soft actuators, including external and integrated approaches. The former with the non‐contacting‐with‐actuator installation, has the advantage of limiting the influence on the motion performance, which is suitable to obtain the information of the entire robotic system, but of less specific feedback of each actuator. For example, camera is utilized to modulate the complex continuous soft surfaces,^[^ ^30^ ^]^ and camera recordings enable the closed‐loop controls for precise underwater manipulations of the soft robotic arms.^[^ ^31^, ^32^ ^]^ The honeycomb pneumatic network arm equips with motion capture system for feedback control.^[^ ^33^ ^]^ The bulky profile, strict setup requirements, and high cost of the external approaches make it difficult to form a compact self‐sensing actuator module, especially for actuator‐level sensing in a multi‐actuator robot.

The integrated approach has the potential to combine the actuation and sensing functional parts into a compact body, enabling actuator‐level motion sensing and allowing easy arrangement of modules in different configurations.^[^ ^34^, ^35^, ^36^, ^37^, ^38^ ^]^ For instance, it is straightforward to embed commercial sensors, such as resistance and capacitive sensors for shape measurement,^[^ ^39^, ^40^, ^41^ ^]^ and strain gauges combined with pressure sensors for force measurement,^[^ ^42^ ^]^ based on task requirements. The pressure‐deformation dual‐modal self‐sensing actuator^[^ ^43^ ^]^ realizes model‐based spatial position and three‐axial force estimations at the joint level, by the sensor fusion of embedded pressure and laser sensors within each actuator. Recently, flexible sensors,^[^ ^44^, ^45^ ^]^ such as electronic skins,^[^ ^46^, ^47^, ^48^ ^]^ have shown great potential for integration with soft actuators, as they are made of similar soft‐material substrates with compatible stretchability. With the integrated approach, the deformation of sensing parts is synchronized with the motion of actuating parts, reducing the morphological discrepancy between the motion path of soft actuators and the perception path when rigid external sensors are used.

However, the approach employs multi‐step integration of the separately manufactured parts, where the additional connections are necessary, hinders the compact design, and introduces deformation differences between attached components, especially in multidimensional motions. Currently, although composite material units fabricated by 3D/4D printing^[^ ^49^, ^50^, ^51^ ^]^ show promising potential to obtain soft substrates integration, non‐single materials in combination generate heterogeneous stresses and strains during synchronous motions induced by the non‐uniform stiffness, leading to complexity in motion sensing. When the actuation and sensing parts rely on different forms of energy conversion principles, the mapping process between them requires additional calculations and calibrations. For instance, the signal differences between pneumatic actuation and non‐pneumatic sensing rely on complex mapping methods such as quantitative analysis models^[^ ^43^ ^]^ and machine learning.^[^ ^52^, ^53^ ^]^

State‐of‐the‐art works utilize pneumatic pressure of the cavity made of single material to realize motions and touch sensing,^[^ ^54^, ^55^ ^]^ in which the pressure‐change feedback is facilitated by the cavity's passive deformation during interaction with the environment. Thus, using single material to construct the structure and pressure sensors to record the changes is a feasible way to realize motion and sensing integration in soft actuators. In our previous works,^[^ ^29^, ^56^ ^]^ a series of soft robotic joints with active and passive actuators in antagonistic configurations were proposed to realize 3‐DOF movements and 1‐DOF sensing. However, few research studies have focused on the multidimensional motions and sensing of integrated compact soft actuator made of a single substrate.

In this work, we explore the multicavity functional possibility for soft pneumatic actuator to integrate multidimensional motions and sensing in a compact structure made of single material.

The main contributions are summarized as follows:

1. Inspired by the constitution of human muscle bundle with the extrafusal and intrafusal muscle fibers, the concept of multicavity functional integration (McFI) for the soft pneumatic actuator is proposed to realize multidimensional motions and embedded sensing by the opened active cavities (for motion and sensing) and the sealed passive cavity (for sensing). The bio‐inspired interweaving foldable endomysium (BIFE) separates the cavities within a cylindrical bellow and serves as a structural reinforcement similar to the separation and supportive efficacy of endomysium in a muscle bundle, regulating the undesirable deformation and allowing adjoint motions of the cavities.
2. The foldable multicavity design method is introduced, realizing desired motion definition, structural reinforcement, and deformation coupling of cavities, enabling one‐step fabrication using single material. To realize airtightness and fabrication of the single‐material foldable multicavity structure, the optimized Fused Dmeposition Modeling (FDM) 3D printing technique is investigated, and the McFI actuator prototypes are produced.
3. The kinematic and sensing models of the McFI actuator are developed and validated by experimental results, hence, verifying the multidimensional motions, including elongation, contraction, and bidirectional bending, and the simultaneous sensing capability, including the perception of spatial position, orientation, and axial force.
4. Two McFI‐actuator‐driven soft robots are developed, namely, the soft crawling robot that locomotes planarly and reconstructs its crawling path, and the narrow‐maneuverable soft gripper with multimodal fingertip operations and interactive sensing of object size, validating the benefit of the McFI actuator in soft robotic applications in achieving versatile perceivable maneuvers without complex linkages or additional sensors.

## 2 Results and Discussion

### 2.1 Concept of 3D‐Printed Multi‐Cavity and Functional Actuator

**Figure 1:**
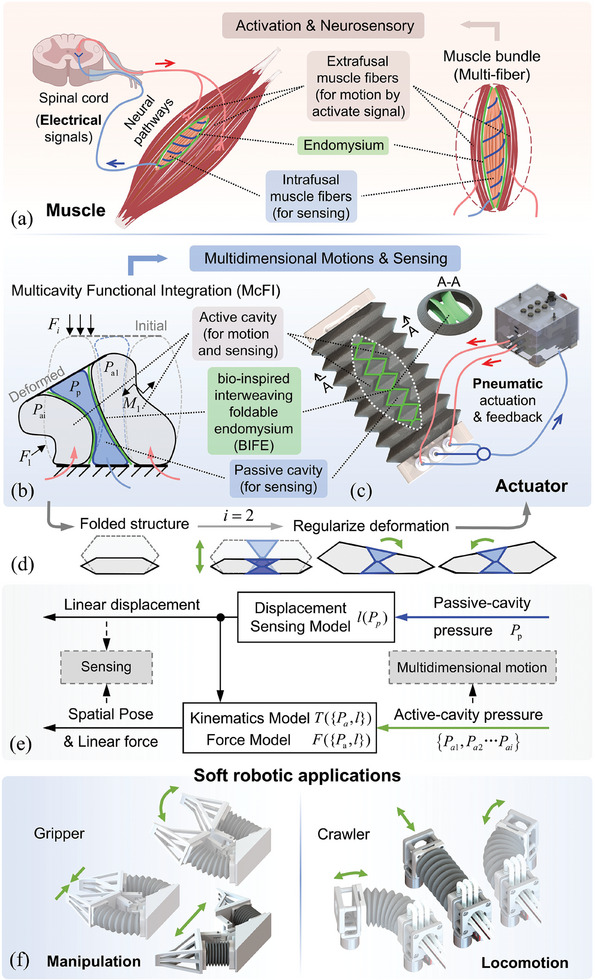
Overview of the proposed concept and the McFI approach for soft actuators integrated multidimensional motions and sensing functionalities. a) The biological multi‐fiber mechanism of the muscle bundle enables simultaneous activation and neurosensory. Inspired by this, b) the McFI approach is proposed to promote c) single‐material actuator to realize both motions and sensing, d) utilizing folded structure and BIFE to regularize the multicavity deformation. e) The kinematic and sensing models for the McFI actuator based on multicavity pneumatic pressures. f) Two robotic demonstrations were constructed, including a soft crawling robot and a narrow‐maneuverable soft gripper.

The concept of McFI involves the actuation and sensing functions into multiple foldable cavities in one compact actuator. It is inspired by the human muscle, as shown in **Figure** **1**a, where the muscle bundle is capable of both activation motions and neurosensory functions due to the presence of extrafusal muscle fibers and intrafusal muscle fibers, which are structurally separated and supported by the endomysium.^[^ ^1^, ^2^ ^]^ The extrafusal muscle fibers actively produce powerful contractions under electrical stimulation, collaboratively executing purposeful movements. During the movements, the intrafusal muscle fibers are passively pulled to produce adjoint deformation, triggering electrical signal changes to achieve neurosensory feedback reflecting muscle movements and forces. The endomysium between the fibers separates the functional parts, maintains the structural integrity, and coordinates with the deformations of fibers.^[^ ^1^, ^57^ ^]^

In the proposed McFI concept, the soft actuator is designed with a multi‐cavitary structure made of a single material. The is designed as the sharing wall of adjacent cavities, dividing and reinforcing the actuator inward to form multiple cavities. Some cavities are kept open and can be actuated with air pressure to actively generate deformation, while others are sealed and passively squeezed following the active deformation of open cavities (Figure 1b). Specifically, these open cavities generate multidimensional motions and actuation forces during actuation, which are reflected in the deformation of both open and sealed cavities passed through via the force transmission of the BIFE. With the passive squeezed deformation of the sealed cavity occurring concomitantly, the pneumatic pressure changes of the active and passive cavities are as feedback can be used to reflect the actuation performance of the McFI actuator. This is based on the physical characteristics of soft actuators where both the forces and displacements relate to the multicavity pneumatic pressure. Therefore, the arrangement of open and sealed cavities enables both actuation and sensing functionalities within a single soft actuator.

Taking a three‐cavitary actuator as an example, two open active cavities and one sealed passive cavity are arranged, with the sealed cavity positioned in the middle and circumferentially surrounded by the open cavities. This configuration produces concomitant deformation of the sealed cavity and reduces the dissymmetry torque effect. Particularly, to guide the irregular deformation of multicavity, mainly caused by external forces and the interaction between adjacent cavities, the folding bellows (Figure 1d) are introduced to regularize the overall motion of the actuator, including elongation, contraction, and plane bidirectional bending motions, while ensuring the perception by multicavity pressure feedback. The three‐cavitary and folding bellow structures contribute to a McFI actuator (Figure 1c), which is fully 3D‐printed using a single material. This McFI actuator is usable alone and easily applied to various soft robotic configurations and connected into existing pneumatic system without additional modification. Based on the multicavity pneumatic pressure of the active and passive cavities, the kinematics model of multidimensional motions and the perception model of spatial position and axial force of the proposed McFI actuator are constructed, as shown in Figure 1e. Furthermore, through the modular splicing of the proposed McFI actuator, various soft robots are easily built with multimodal operations and perceptions, among which two robotic examples are demonstrated to showcase the application potential in Figure 1f, including a soft crawling robot with trajectory reconstruction for planar locomotion and a narrow‐maneuverable soft gripper with multiform fingertip manipulation and exteroception.

### 2.2 Modeling and Design of McFI Actuator

**Figure 2:**
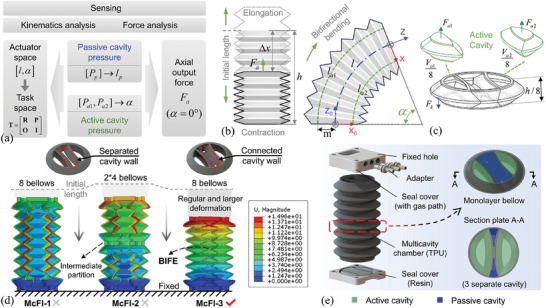
Modeling and design of the proposed McFI actuator. a) The derivation process of the kinematic and sensing models for spatial pose and axial force based on the multicavity pressure feedback. b) The kinematic analysis diagram showing multidimensional motion and sensing of spatial position and orientation. c) The force analysis diagram of the McFI actuator during linear motion. d) Strain simulations of different designs with McFI‐1, McFI‐2, and McFI‐3 were carried out to reveal the multicavity‐deformation effect during actuation, where the optimal McFI‐3 serving as the design paradigm. e) The constructed prototype of the McFI actuator.

The multidimensional motions, axial output force, and sensing are related to the pneumatic pressure changes in the open and sealed cavities of the proposed McFI actuator. Therefore, it is essential to establish these relationships among actuator kinematics, forces, and multicavity pressure. These relations and entire workflow of the self‐sensing estimation are illustrated in **Figure** **2**a. First, based on the constant amount of gas in the sealed cavity, the mapping relationship between passive‐cavity pressure and the actuator's length *l* is investigated, where the displacement is induced by passive‐cavity deformation concomitant with the actuation motions and represents the length's changes. Then, combined with the kinematics analysis of active‐cavity actuation, the relationship between two active‐cavity pressures and bending angle α is derived, which further combines the above‐mapped length to establish the relationship from multi‐cavity pressure feedback to the actuator space [*l*,   α] of multidimensional motions. This relationship enables mapping spatial position and orientation in the task space from multicavity pressure feedback, with a derived mathematical transformation matrix T. Additionally, During the McFI actuator execute linear motions without bending, two active‐cavity pressures can be utilized to calculate the actuation work done, which is divided into two parts, one for generating the actuator's linear displacement and the other for output force. Therefore, combined with the estimated length from passive‐cavity pressure feedback, the relationship from multi‐cavity pressure feedback to axial displacement and output force of the McFI actuator is established by force analysis based on the law of energy conservation. This relationship enables axial force‐displacement simultaneous perception of the McFI actuator by multicavity pressure feedback.

Based on the described workflow, we derived the corresponding models for sensing. Regarding the passive‐cavity deformation, the pressure changes are directly related to the volume change when the amount of gas is constant. According to the ideal gas law, i.e., *PV* = *nRT*, the pressure is inversely proportional to the volume. Therefore, the passive‐cavity pressure before and after deformation can be characterized as follows:

(1) $$\begin{array}{c} \left(P_{p}+P_{AT}\right)V=\left(P_{p0}+P_{AT}\right)V_{0}\\ V=\bar{S_{p}}\left(h+\Delta x\right),\;V_{0}=\bar{S_{p}}h \end{array}$$

where *P_p_* and *V* are the real‐time pressure and the post‐deformed volume of the passive cavity, respectively; *P*_*p*0_ and *V*_0_ are the initial pressure and volume; *P_AT_* is the atmospheric pressure; $\bar{S_{P}}$ is the equivalent cross‐sectional area; while *h* and Δ*x* are respectively the initial length and displacement of the McFI actuator, as shown in Figure 2b.

Assuming the passive‐cavity adjoint motion is regular, the equivalent cross‐sectional area is considered constant, and the passive‐cavity pressure change is only related to the displacement. So that the passive‐cavity pressure feedback can be utilized to accurately estimate the actuator length, with the developed relationship as follows:

(2) $$\frac{P_{p0}+P_{AT}}{P_{p}+P_{AT}}-1=\varepsilon_{V}\frac{\Delta x}{h}$$

where ε_*V*_ is a correction factor for the deformed volume, which can be obtained experimentally by linear fitting. Thus, the explicit function for mapping the passive‐cavity pressure to actuator length *l* can be further developed as follows:

(3) $$l=\frac{h\left(P_{p0}-P_{p}\right)}{\varepsilon_{V}\left(P_{p}+P_{AT}\right)}+h$$

assuming the actuator length is equal to the passive‐cavity length with *l* = *l_p_* = Δ*x* + *h*.

In order to further obtain the bending angle α of actuator space, we deduced the relationship between two active‐cavity pressures and the bending angle through kinematic analysis. Based on the constant curvature hypothesis,^[^ ^58^ ^]^ the result is developed as follows:

(4) $$\alpha =\left[l_{a1}-l_{a2}\right]/2m=\left[f_{xa}\left(\Delta P_{a1}\right)-f_{xa}\left(\Delta P_{a2}\right)\right]/2m$$

where *l*_*a*1_ and *l*_*a*2_ are the central arc lengths of two active cavities after actuation, respectively. *m* is the central distance between either of the two active cavities and the actuator. Particularly, the function *f_xa_* (Δ*P_a_*) represents the transformation Equation from the actuated pressure difference Δ*P_a_* to the active‐cavity deformed length Δ*l_a_*. This function for targeted McFI actuator prototype can be obtained through experimental characterization and data fitting. Therefore, here we have established the sensing model of the actuator space [*l*,   α] from the pressure feedback of both the passive and active cavities.

Furthermore, to estimate the spatial orientation and position (*x*, *y*, *z*) of the end of the McFI actuator, the actuator space [*l*, α] is mapped to the task space, obtaining the transformation matrix **T** with rotation matrix **R** and position matrix **P** = [*x*, *y*, *z*]^*T*^ as follows:

(5) $$T=\left[\begin{array}{cc} R & P\\ O & I \end{array}\right]=\left[\begin{array}{cccc} \cos\alpha & 0 & \sin\alpha & l/\alpha \left(1-\cos\alpha \right)\\ 0 & 1 & 0 & 0\\ -\sin\alpha & 0 & \cos\alpha & l/\alpha \sin\alpha \\ 0 & 0 & 0 & 1 \end{array}\right]$$

which is convenient for the analysis of applications in soft robots.

As for the axial force analysis (Figure 2c) during linear motions of the McFI actuator with Δ*P*_*a*1_ = Δ*P*_*a*2_, the work done by the active‐cavity pneumatical actuation was divided into two parts: one parts produce the actuator's displacement sensed by the passive‐cavity pressure feedback, and the other converts to output force. Thus, we can obtain:

(6) $$F_{a1}+F_{a2}=\Delta P_{a1}\varepsilon_{S}\bar{S_{a1}}+\Delta P_{a2}\varepsilon_{S}\bar{S_{a2}}=f_{k}\left(\Delta x\right)\Delta x+F_{a}$$

where $\bar{S_{a1}}$ and $\bar{S_{a2}}$ are respectively the equivalent cross‐sectional areas of the two active cavities, and can be characterized by the ratio of the active‐cavity volume *V_a_* to the initial length *h*, with the same conditions within the actuator prototype. ε_*S*_ is the area correction factor of these active cavities. The function *f_k_* (Δ*x*) represents the axial stiffness of the McFI actuator with a sealed passive cavity, which is directly related to the displacement Δ*x*.

Then, by substituting Equation (3) into Equation (6), the focused axial output force *F_a_* can be developed as:

(7) $$F_{a}=2\varepsilon_{S}\bar{S_{a1}}\Delta P_{a1}-f_{k}\left(\left[\frac{h\left(P_{p0}-P_{p}\right)}{\varepsilon_{V}\left(P_{p}+P_{AT}\right)}\right]\right)\frac{h\left(P_{p0}-P_{p}\right)}{\varepsilon_{V}\left(P_{p}+P_{AT}\right)}$$

This Equation establishes the mapping relationship form multicavity pressure feedback to the axial output force, where other parameters are determined by the design and characteristics of the targeted actuator prototype. Thus, we can synchronously estimate the displacement and force based only on the multicavity pneumatic signals, even when the McFI actuator encounters to unknown obstacles and produces unexpected deformations or interactions.

To integrate multidimensional actuation motion and sensing functionalities within a single soft actuator, the primary focus of the proposed multicavity design is on the BIFE. The BIFE serves as shared walls and plays a key role not only in separating cavities, but also in transmitting the deformation from the open cavity to the sealed cavity for functional integration. Therefore, besides maintaining airtightness between the open and sealed cavities, it is crucial to realize the concomitant deformation of the sealed cavity during actuation and ensure its capability for sensing functions. In the section that follows, the McFI actuator's design is proposed, optimized, fabricated, and evaluated for its functional performance and the influence of key design parameters through both simulations and experiments.

A Finite Element Method simulation was conducted on chamber designs McFI‐1, McFI‐2, and McFI‐3, as shown in Figure 2d. Each design features two open cavities for spatial bidirectional and linear movements, and a sealed cavity dedicated for accompanied deformation. The primary difference among these designs lies in the constraints applied to the sealed‐cavity walls, which may potentially impact the desired performance. The cavities in McFI‐1 are separated by two distinct thin walls without an interweaving connection, where both the open and sealed cavities share the independent wall. The strain in the shared walls has been analyzed, as shown in Figure 2d. It is observed that unwanted radial strains occur in these shared walls under pneumatic actuation. This unwanted radial strain may be due to the unconstrained radial freedom and excessive compliance of the large‐area shared cavitary wall, potentially causing irregular deformation and functional failure of sensing during movements. To address this issue, constraints have been introduced to the shared walls in two ways: radial constraints in the middle of the actuator, as in McFI‐2, and the BIFE with interweaving connected constraints in every layer of the actuator, as in McFI‐3. The simulation results of these two constrained actuators are also shown in Figure 2d. The former form slightly weakens the work‐loss defect by decreasing the curved areas of the shared cavity wall, but loses foldability of connection and does not fundamentally improve performance, as the separated cavity wall is similar to the compared McFI‐1 form. In contrast, the latter form significantly improves the transfer of the actuation work done to more uniform and larger regular deformation within the targeted axial direction, while maintaining overall foldability, due to the interweaving foldable design of the proposed BIFE structure. Thus, the McFI‐3 form is chosen as the design paradigm.

The complete structure of the McFI actuator is illustrated in Figure 2e, consisting of a 3D‐printed multicavity chamber, end covers with built‐in gas paths, and corresponding‐quantity tracheal adapters, which are tightly bonded together with glue (YH‐888, YIHE inc.) to ensure the junction's airtightness. The multicavity chamber is made up of eight layers of bellows containing three cavity structures, with two active and one passive cavities, sharing adjacently the cavitary wall of BIFE.

### 2.3 Fabrication and Characteristics of McFI Actuator

**Figure 3:**
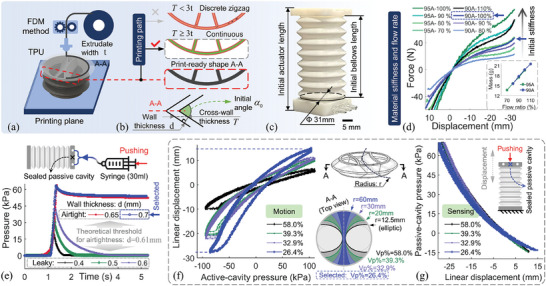
Fabrication and design consideration of the multicavity chamber with BIFE. a) Based on the FDM 3D printing method, b) the crucial printing paths were analyzed and regulated to ensure the multicavity chamber's airtightness. c) The McFI actuator prototype was developed. d) The influence of the material stiffness and extrudate flow rate on prototype stiffness. e) The comparison of the printed McFI actuator's airtightness designed with different wall thicknesses. The influence evaluation of the multicavity volume ratio on the McFI actuator's functional performance, especially on f) the motions and g) sensing.

Directly related to the critical design parameters of the actuator's thickness and multicavity volume ratio, and the 3D printing settings, the airtightness and structural stiffness are primary considerations when fabricating the actuator chamber, which has a single‐material foldable complex‐multicavity structure. In this case, we utilized the FDM printing method to produce the single‐material complex chamber with Thermoplastic Polyurethane (TPU), as illustrated in **Figure** **3**a. Key printing parameters, covering the printing path and the extrudate flow rate, were taken into account during the fabrication process to ensure airtightness. Specifically, the multicavity chamber is cut horizontally and printed layer by layer, where each layer structure is composed of a stack of extrudates along the printing path. Based on the layer slice as Figure 3b, the printing path exhibits a discrete zig‐zag pattern with discontinuous padding when the cross‐wall thickness *T* is less than three times the extrudate width *t*, i.e., *T* < 3*t*, which leads to weak airtightness and potential stress concentration. Therefore, to obtain a continuous printing path and avoid this defect, the McFI actuator's wall thickness *d* and the extrudate width *t* must satisfy the following geometric relationship:

(8) $$d\geq 3t\sin\left(\alpha_{0}/2\right)$$

where α_0_ is the initial dihedral angle of the bellows. Thus, we preliminarily determined the specific design parameters for the McFI actuator prototype, as shown in **Table** **1**. The detailed 3D printing settings for manufacturing are shown in **Table** **2**.

**Table 1:** Geometry parameters of the McFI actuator prototype.

Geometry parameter	Value	Geometry parameter	Value
Initial bellow heigh *h*	68 mm	Initial dihedral angle α_0_	73°
Wall thickness *d*	0.7 mm	Central distance *m*	12 mm
Inner and outer bellow diameter	31 mm 40 mm	Three cavities’ volume *V*_*a*1_: *V_p_*: *V*_*a*2_	(100 mm^3^) 212.4:152.6:212.4

**Table 2:** 3D printed optimized parameters for TPU‐based prototype of airtight multicavity.

Geometry parameter	Value	Geometry parameter	Value
Extrusion width *t*	0.34 mm	Default printing speed	15 mm s^−1^
Single layer heigh	0.1 mm	X/Y/Z Axis movement speed	50 mm s^−1^ (X/Y) 5mm s^−1^ (Z)
Retraction length	0.5 mm	Retraction speed	60 mm s^−1^
Infill shape	Linear	Printing temperature	220 °C
Start‐point type of each layer	Minimum distance	Heat bed temperature	28 °C
Start‐point location	Dip/bump angle	Fan speed	100%

The constructed McFI actuator prototype is shown in Figure 3c. Then, the influence of the material hardness and extrusion flow rate on the fabricated actuator's stiffness was investigated. Following the aforementioned design and printing parameters, eight McFI actuators were fabricated using material hardness ranging from 90 to 95A and extrusion flow rates between 70% and 110% for comparison. The relationships between forces and axial displacements are shown in Figure 3d. The results indicate that the prototype's physical stiffness, represented by the curve's slope, decreases when the TPU material hardness is reduced from 95 to 90 A or the percentage of the extrusion flow rate is reduced. Here, to achieve a balance between stiffness and flexibility, we utilized 90 A TPU material and 100% extrusion flow rate for fabrication, which were employed to construct the McFI actuator prototype.

On this basis, to validate the proposed geometric Equation (8) and reveal the impact of the actuator's wall thickness *d* on airtightness, five McFI actuator prototypes with different wall thickness are produced, including 0.4, 0.5, 0.6, 0.65, and 0.7 mm. Among them, the theoretical threshold of wall thickness for airtightness is calculated as 0.61 mm based on Equation (8), which is covered by the setup experimental wall‐thickness span. Successively, we set up a syringe with a 30 mL volume and pushed it fully to pressurize each prototype, and then maintaining the pressurized state for some time. The pressure changes of these prototypes were recorded during the process, as shown in Figure 3e. The results indicate these prototypes with a greater wall thickness than the theoretical threshold have stronger airtightness, which has the continuous printing path of the 3D printing slice, maintaining pressure stable after pressurization. In turn, the other prototypes showcase a weaker airtightness where their pressure continues to decay during the static stage until they leak completely, and the 3D‐printed prototype's leaks faster with a smaller wall thickness indicating an even weaker airtightness. This phenomenon proves the effectiveness of the considerations on the 3D printing path in Figure 3b and further validates the proposed Equation (8) for ensuring the McFI actuator's airtightness. Here, we select a wall thickness of 0.7 mm for design.

Additionally, the impact of the passive‐cavity volume ratio on the McFI actuator's functional performance was evaluated, especially on the motion and the integrated sensing feedback. By adjusting the radius and contour of the sharing wall between adjacent cavities, four McFI actuator prototypes were designed for comparison experiments, with a similar interweaving BIFE structure but different passive‐cavity volume ratio *V_p_*% of 26.4%, 32.9%, 39.3%, and 58.0%, as shown in Figure 3f. For the linear motion performance during reciprocating pneumatic actuation, as shown in Figure 3f, the result indicates that the motion range decreases with the ratio increases within the same actuation‐pressure range, which may be restricted by the increased stiffness brought by the increased sealed volume of the passive cavity and the decreased actuation work by the reduced active‐cavity volume. As for the sensing feedback performance, especially on the passive‐cavity pressure feedback for displacement sensing, each prototype is pushed axially by external force to execute three reciprocating displacements with the active cavity opened. The real‐time displacement and corresponding passive‐cavity pressure feedback were recorded, as shown in Figure 3g. The result shows the same trend and similar values of feedback in each prototype, although with different volume ratios. This phenomenon matches the revealed relationship by Equation (3), where derived the direct mapping relationship from the deformation to the passive‐cavity pressure regardless of the cavity volume. Therefore, to obtain a larger motion range, the multicavity volume ratio of the McFI actuator was designed with the passive‐cavity volume ratio of 26.4%, which parameter is displayed in Table 1.

**Figure 4:**
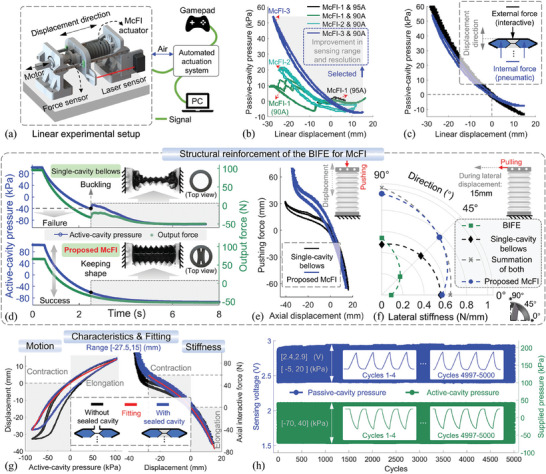
Characterization of the McFI actuator. a) A schematic diagram of the linear experimental setup. b) The passive‐cavity pneumatic pressure feedback from the McFI actuator with different designs was compared to validate the strain simulation. c) The comparison of passive‐cavity pressure feedback caused by different force inducements of the external interaction and internal pneumatic actuation. The experimental validation of the BIFE's structural reinforcement for the McFI actuator, including d) the pressure‐bearing performance, e) axial stiffness, and f) lateral stiffness. g) The characterization comparison of the McFI actuator's motion and stiffness with the passive cavity open versus sealed, along with the fitted characteristic equations. h) An endurance test of the McFI actuator involving repeated motion and simultaneous sensing feedback.

So far, the parameters of the structural design and 3D‐printed fabrication for the McFI actuator have been clarified and optimized, with the experimental validation. Specifically, the key to the sensing functions based on the multicavity pressure feedback lies in validating the relationships among multicavity pressure, actuator motion, and force, where the motion involves linear and spatial cases. Here, the linear experimental setup is shown in **Figure** **4**a, utilizing the lead‐screw slide‐rail device with various commercial sensors for linear motion testing of characteristic calibration, as used in previous work.^[^ ^24^ ^]^ In this device, the tested McFI actuator can be mounted and pneumatically modulated by the automatic actuation system to perform linear displacement along with the slide‐rail constraint, while the multicavity pressure feedback and motion's ground truth can be captured in real‐time and further uploaded to the MCU and PC for processing. On this basis, the actuator prototypes of McFI‐1, McFI‐2, and McFI‐3 were fabricated and utilized to further verify the aforementioned simulation results. Figure 4b presents the experimental relationship between the linear displacement and passive‐cavity pressure changes during elongation and contraction as the active cavity is pneumatically actuated. The results showcase a phenomenon similar to the above simulation that the prototypes of McFI‐3 form exhibit regular and highly repeatable feedback to map the synchronous displacement. Thus, the sealed passive‐cavity pressure could be utilized to quantitatively indicate the displacement variation of the McFI actuator. In contrast, the prototypes of McFI‐1 and McFI‐2 forms present random pressure variations with displacement changes owing to the aforementioned unwanted wall deformation.

Notably, this perceived displacement can be caused not only by the active pneumatic regulation of internal actuation force but also by external force such as interaction. To investigate the potential impact of this phenomenon on feedback, we conduct the comparative test of passive‐cavity pressure feedback under different force inducements. In the case caused by an external force, the McFI actuator opens the active cavity to engage the atmosphere and generates linear motion by being pulled through the motor‐driven screw, while the passive‐cavity pressure feedback is recorded in real‐time. Figure 4c shows a similar feedback trend and value in both cases with different inducements. Therefore, this feedback from passive‐cavity pressure is less affected and more effective in estimating displacement even with the unknown interaction, compared to the model‐based non‐closed loop method,^[^ ^24^, ^59^ ^]^ which cannot ignore the interference of external force.

Additionally, this multicavity mechanism with sealing specific cavity inevitably introduces changes in motion performance. Benefiting from the foldable design and the embedded BIFE structure, the McFI actuator enables the structural reinforcement while ensuring sufficient movement range with less motion attenuation. Here, we evaluate the BIFE's structural reinforcement effort for the McFI actuator, the influence of sealing passive cavity on the actuator's motion performance and physical stiffness, and fit the displacement function *f_xa_* (Δ*P_a_*) and stiffness function *f_k_* (Δ*x*) for the above perception model within the sealed state of the passive cavity.

For the BIFE‐separated multi‐cavity structural reinforcement effect, we comprehensively investigated it from three aspects, compared the McFI actuator with the single‐cavity actuator without BIFE, including: 1) the pressure‐bearing enhancement for avoiding structural buckling, 2) the rising axial stiffness, and 3) the enhanced lateral stiffness, as shown in the added Figure 4d–f respectively. Specifically, the single‐cavity actuator has the same outer contour of the McFI actuator and was fabricated with the same settings.

For the pressure‐bearing capability, we initially fixed the ends of both the single‐cavity actuator and the McFI actuator to limit their axial displacement and pressurized them to the same positive pressure. Then, both of them were depressurized continuously, while their actuation pressure and output force were simultaneously recorded in real‐time, as shown in Figure 4d. The result indicates that the single‐cavity actuator displays buckling when the pressure is below −40 kPa, which affects its predefined motion. In contrast, the McFI actuator successfully maintains the structural stability spanning from −100 to 100 kPa without buckling. Notably, the output force when the buckling occurred represents the actuation‐energy the actuator is able to withstand, which characterizes their structural strength. Therefore, the results validate the structural reinforcement for the McFI actuator to own greater pressure‐bearing capability and structural stability. In the evaluation of the axial stiffness, we fixed the bottom of these prototypes on the linear experimental setup and drove the screw to push their top at a constant speed for the forced displacement. During this process, the pushing force and the axial displacement were recorded in real‐time, as shown in Figure 4e, in which the slope represents the axial stiffness. The results show that the McFI actuator significantly improves the axial stiffness, especially in contraction. For the lateral‐stiffness comparison, we fixed the bottom‐end of these prototypes and a bare BIFE structure cut from a McFI actuator, while driving the screw to pull their top‐end in the lateral direction at a constant speed. The lateral stiffness is represented by the ratio of the pulling force and lateral displacement, while the lateral stiffness at the 15 mm displacement was calculated. The tests cover three orientation cases of 0°, 45°, and 90° for these prototypes, as shown in Figure 4f. The results indicate that the lateral stiffness of the McFI actuator is significantly reinforced over the single‐cavity actuator, almost equal to the superposition of the single‐cavity actuator and the BIFE structure.

Additionally, to characterize the influence of sealing specific cavity on the McFI actuator's motion and physical stiffness and further fit the function, two cases of the McFI actuator were set up for experiments, an opened and a sealed passive cavity. For motion characterization, both cases are pneumatically actuated to generate linear motion along the slide‐rail device, while the relationship between actuation pressure and displacement is recorded to reveal their motion performance. For stiffness characterization, the experimental process of both cases is similar to the previous axial stiffness test. As shown in Figure 4g, after sealing the passive cavity, the primary influence occurs during the contraction phase, which shows a slightly reduced motion range with higher stiffness. The prototype characteristics within the effective motion range [− 27.5, 15](*mm*) are fitted as follows:

(9) $$\begin{array}{c} f_{xa}\left(\Delta P_{a}\right)=4.7\times 10^{-6}\Delta {P_{a}}^{3}-1.1\times 10^{-3}\Delta {P_{a}}^{2}+0.2\Delta P_{a}\\ f_{k}\left(\Delta x\right)=1.37\times 10^{-3}\Delta x^{2}+0.08\Delta x+3.27666 \end{array}$$

The repeatability and durability of the McFI actuator were further investigated. In this test, the actuator underwent a cycle of elongation and contraction under a preprogrammed actuation pressure from −70 to 40 kPa over a period of 2 s. We synchronously recorded the pressure values for both open and sealed cavities. The results of 5000 cycles in Figure 4h indicate that the McFI actuator maintains high repeatability of motion performance and stable multicavity pressure feedback, without air leakage or performance degradation, demonstrating its long‐term reliability and application potential.

### 2.4 Multidimensional Motion and Functional Validation of McFI Actuator

The McFI actuator is capable of linear contraction, extension, and 2D bending movements. During these motions, the pressure feedback of the sealed passive cavity is utilized to measure the actuator's central length. Combining the actuation pressures from two active cavities into models (4) and (6), we can further determine both the bending movements and axial forces from these multicavity pressure feedbacks. This section will validate the McFI actuator's capabilities on multidirectional motions and integrated sensing functionalities, covering linear motion and spatial motion with bending, together with the axial displacement‐force simultaneous perception.

**Figure 5:**
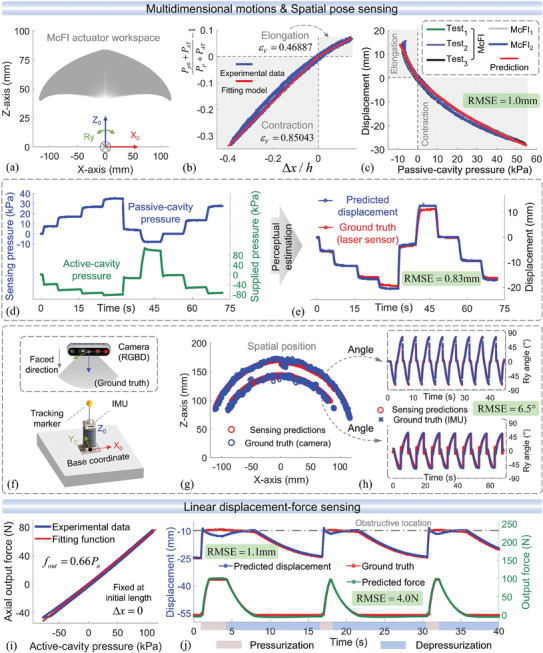
Experimental validation of multidimensional motions and integrated sensing. a) The effective workspace of motion and sensing of the McFI actuator. b) Experimental characterization and coefficient fitting for the actuator‐length sensing function. c) The validation of displacement sensing with multiple McFI actuator prototypes. During the static‐state sensing validation, d) the multicavity pressure of both active and passive cavities in the process, and e) the simultaneous sensing estimation compared with the ground truth were presented. f) The spatial‐motion experimental system for obtaining ground truth of the spatial position and orientation. g) The experimental validation of sensing spatial position during two preprogrammed arc‐trajectory motions, with h) the comparison of the real‐time bending angle estimation and ground truth during these dynamic processes. i) The characterization and coefficient fitting of the McFI actuator output‐force performance. j) The validation of simultaneous displacement‐force sensing during linear motion with unknown interactions by obstruct.

Following the kinematic model (5), the workspace of the McFI actuator is illustrated in **Figure** **5**a. Within this workspace, the displacement‐sensing model (2) was first verified using the transformed experimental data (shown in Figure 4c) of the linear displacement and the corresponding passive‐cavity pressure, as shown in Figure 5b. The results indicate a linear prediction trend consistent with Equation (2), with different slopes during elongation and contraction. In this Equation, the volume correction factor, presented by the curve slope, is experimentally obtained for the length‐sensing Equation (3), as follows:

(10) $$\varepsilon_{V}=\left\{\begin{array}{c} 0.46887,\;\;\mathrm{Elongation}\\ 0.85043,\;\;\mathrm{Contraction} \end{array}\right.$$

To verify the effectiveness of the length‐sensing model with Equation (3), evaluate the consistency of the 3D‐printing fabrication, and validate the repeatability and accuracy of the self‐sensing function, three actuator prototypes of McFI, McFI_1_, and McFI_2_, with identical design and fabrication process, were constructed and tested. They were installed on the linear experimental setup and pneumatically actuated to perform repetitive reciprocating motion three times in each group test. The McFI_1_ and McFI_2_ were tested in one group test, respectively. the McFI prototype was tested in three group tests. During these tests, the passive‐cavity pressure feedback was recorded to further estimate the sensed displacement simultaneously. This estimated displacement was compared with the ground truth, as shown in Figure 5c. The results show the high repeatability and accuracy of the self‐sensing function by comparing the test results of McFI, and the fabrication consistency by comparing these different prototypes. The Root Mean Square Error (RMSE) between the predicted and actual displacement of three McFI actuators is 1.0 mm, highlighting the precise performance of the integrated sensing function.

Apart from the validated displacement perception, the static‐sensing performance is of equal importance for evaluating the integrated sensing performance. Here, we adopt intermittent depressurization and pressurization to discretely actuate the McFI actuator for performing linear motions, in which the active‐cavity pressures of each step are randomly adjusted between −83 and 100 kPa and maintained for some time at each static stage after actuation. The entire process underwent nine steps of static stages with different actuation pressures and durations, taking 72 s in total. During this process, the multi‐cavity pressure feedback of the McFI actuator was recorded in real‐time, including the active‐cavity and passive‐cavity pressure, as shown in Figure 5d. Simultaneously, the predicted axial displacement was calculated from the passive‐cavity pressure feedback, compared with the ground truth by a laser sensor, as shown in Figure 5e. The result shows a RMSE of 0.83 mm between the perception and the ground truth, exhibiting the precise static sensing performance with a smaller error than the displacement‐sensing tests in Figure 5c, which benefit from the stable passive‐cavity pressure feedback at the static stages.

To further verify the sensing model for perceiving spatial movement, combining linear motion and bidirectional bending, an experimental platform was constructed, as shown in Figure 5f, to obtain real‐time spatial‐pose ground truth for comparison with model‐based estimations. In this platform, the testing McFI actuator was fixed at the bottom and mounted with an IMU for getting the ground truth of the orientation and a tracking marker at the top, while a camera was set up to capture the ground truth of the spatial position. Specifically, the McFI actuator repeatedly performed the arc‐trajectory motions with two different paths and dynamically sensed its spatial position and orientation based on the multicavity pressure feedback. The result shown in Figure 5g exhibits the dynamic spatial sensing capability of the McFI actuator with precise position estimation and trajectory reconstruction. During the motion process, the temporal comparison of the bending angle between the perceptive estimation and the corresponding ground truth was illustrated in Figure 5h, showing the precise real‐time dynamic sensing capability for spatial orientation with a high coincidence. The RMSE of the bending‐angle perception is 6.5°. This orientation‐sensing error increases when the bending angle approaches the zero or extreme‐value positions, which may be induced by the hysteresis of the soft‐material substrate.

The above validation and discussion show that the passive‐cavity pressure feedback can be utilized to effectively sense the McFI actuator's central length based on the Equation (3), and further predict the spatial‐motion position and orientation in combination with the active‐cavity pressure feedback. Combining with the length‐sensing Equation (3) and force‐sensing Equation (7), the displacement and axial force of the McFI actuator can be effectively sensed simultaneously from the multicavity pressure feedback during linear motion, even with the unknown interaction. To validate this linear displacement‐force sensing performance, we first experimentally fitted the corrected area $\varepsilon_{S}\bar{S_{a1}}$ for the active cavity to quantify Equation (7). By mounting the McFI actuator on the linear experimental device and constraining its movement with Δ*x* = 0 *mm*, the pneumatic‐actuation energy is fully transferred to output force, and Equation (7) is simplified to $F_{a}=2\varepsilon_{S}\bar{S_{a1}}\Delta P_{a1}$. During reciprocating pressurization and depressurization, the actuation pressure and output force are recorded and shown in Figure 5i. Thus, the corrected area represented by the curve slope can be fitted as $\varepsilon_{S}\bar{S_{a1}}=0.33\left(mm^{2}\right)$. On this basis, we verify the simultaneous sensing performance of the proposed displacement and force sensing models. Specifically, the McFI actuator was fixed on the slide‐rail device (Figure 3e) to perform linear motion freely, while an obstacle was anchored at a distance of − 10*mm* from the actuator's top to block its movement and generate interaction force. The McFI prototype was initially set at ultimate contraction and then the preprogrammed reciprocating pressurization and depressurization were repeatedly performed. During the pressurization process, the prototype elongates until it interacts with the preset obstacle, and then the output force increases from zero. During the subsequent depressurization process, inversely, the force decreases back to zero as the interaction is undone, and then the prototype contracts back to its initial position. Throughout this repeated process, predictions of the sensing displacement and force were calculated based on the multicavity pressure feedback, and the corresponding real‐time ground truth was recorded for comparison. These results shown in Figure 5j demonstrate high accuracy and fast response of displacement‐force simultaneous sensing, with *RMSE* = 1.1*mm* of displacement sensing and *RMSE* = 4.0*N* of force sensing, even in the presence of unknown external interaction.

### 2.5 Demonstrations on Soft Crawling Robot

Benefiting from the functional integration of multidimensional motions and sensing provided by the proposed multicavity mechanism, the McFI actuator can be applied to various soft robotic configurations as a universal stand‐alone actuator module, promoting intelligent soft robotic innovation for wider applications. Here, we present two soft robotic examples to demonstrate the McFI actuator's multifunctionality in robotic applications, including a soft crawling robot for plane‐motion locomotion with crawling‐path sensing and reconstruction, and a narrow‐maneuverable soft gripper for multimodal manipulations with interaction‐object exteroception.

**Figure 6:**
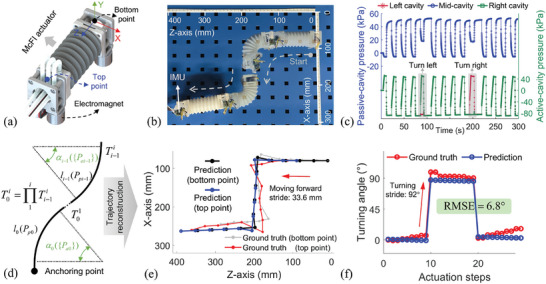
Demonstrations of the soft crawling robot. a) The robot was constructed using a single McFI actuator, b) executed multidirectional motion for a plane Z‐shaped trajectory. c) The multicavity pressure of both active and passive cavities in this crawling process was shown. d) The schematic diagram of the trajectory‐reconstruction workflow for this soft crawling robot. e) The crawling trajectory of this process was reconstructed by each step's sensing estimation and compared with the ground truth. f) The comparison between the real‐time orientation sensing and ground truth during this crawling process.

The constructed soft crawling robot is shown in **Figure** **6**a. The robotic body is equipped with one McFI actuator to enable omnidirectional movement on the plane, including moving forward and backward, turning left and right. Through the alternating anchoring action of electromagnets assembled on the bottom and top points, along with the directional motion of the McFI actuator, this soft crawling robot demonstrates a preset Z‐shaped trajectory on a plane, as shown in Figure 6b and Movie S1 (Supporting Information). During this crawling process, the multicavity pressure was recorded as the feedback for self‐sensing calculation of the actuator and further crawling‐path reconstruction of the robot, including the active‐cavity and passive‐cavity pressure as shown in Figure 6c. The schematic diagram of the trajectory‐reconstruction workflow for this soft crawling robot is presented in Figure 6d and elaborated in detail below. Based on the obtained multicavity pressure feedback, especially the feedback within each static stage when the actuation pressure is stable, the soft robotic motion of each crawling step was estimated. The motion of step *i* consisted of the central length *l*_*i* − 1_ and turning angle α_*i* − 1_, which are calculated by the passive‐cavity and active‐cavity pressure feedback through Equation (3) and (4), respectively. Then, the transformation matrix of each crawling step can be constructed based on Equation (5) and the dimensions of anchoring structures. The full‐process trajectory is calculated by the matrix product of each step $T_{0}^{i}=\prod_{1}^{i}T_{i-1}^{i}$, where $T_{i-1}^{i}$ presents the transformation matrix of crawling step *i*. Through this workflow, the trajectory reconstruction of the plane movement is realized solely using the multicavity pressure feedback, as shown in Figure 6e. The reconstruction result exhibits high credibility compared to the ground truth obtained from the tape measure in video recording. Additionally, the estimated real‐time orientation of each step is shown in Figure 6f, which exhibits a high coincidence compared to the ground truth obtained by the equipped IMU, with an RMSE of 6.8°. These results validate the effectiveness and accuracy of the crawling sensing and trajectory reconstruction from the multicavity pressure feedback and show the crawling performance with the mean crawling forward stride of 33.6 mm and the turning‐angle stride of 92°. This multidimensional crawling capability with trajectory sensing and reconstruction promotes the exploration potential of soft crawling robots in complex environments, achieved by the simple assembly of the McFI actuator without the need for additional sensing systems.

### 2.6 Demonstrations on Narrow‐Maneuverable Soft Gripper

**Figure 7:**
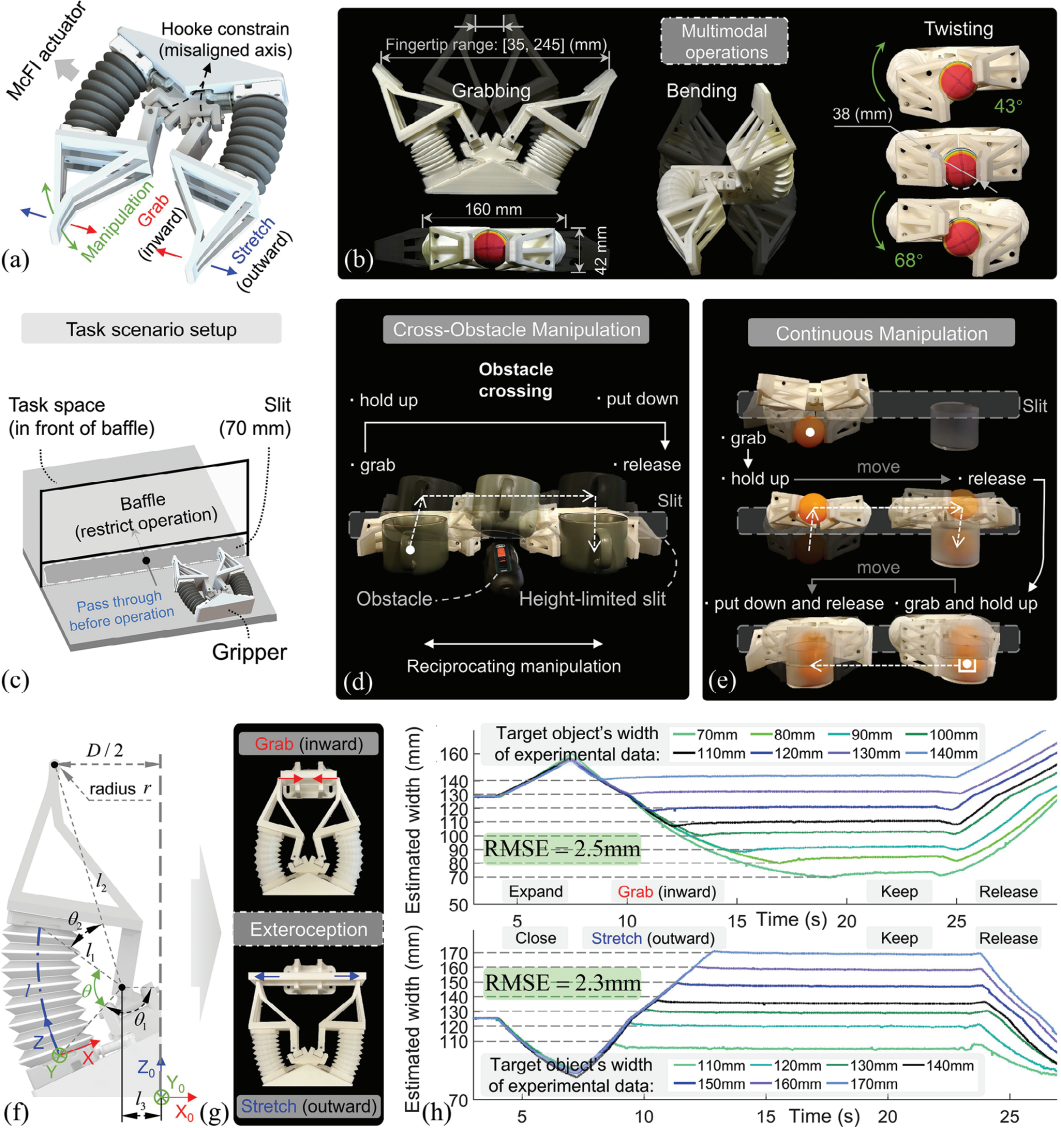
Demonstrations of the narrow‐maneuverable soft gripper. a) The two‐finger soft gripper constructed by the McFI actuator b) exhibits multimodal operations capabilities. c) The experimental setup with baffle and slit for narrow‐environment tasks to verify the developed capability and application potential of this soft gripper, setting two tasks: d) cross‐obstacle manipulation and e) continuous manipulation of two objects. f) The kinematic analysis based on the McFI actuator's sensing model is developed to enable the gripper's exteroception g) for estimating the width of interacted objects during inward grabs and outward stretches. h) A series of grabbing and stretching experiments were conducted to assess the exteroception performance for objects with widths ranging from 70 to 170 mm.

The functional integration of multimodal operation and sensing of the proposed McFI actuator is also demonstrated in manipulation application. Here, we developed a two‐finger narrow‐maneuverable soft gripper, as shown in **Figure** **7**a, where each finger is actuated one McFI actuator and equipped with the Hooke constraint. This enables multimodal fingertip operations integrated with the exteroception to manipulate objects and sense objects’ size. Specifically, the developed gripper exhibits various manipulation capabilities (Figure 7b), including closing fingers for grabbing objects inward, extending fingers for stretching objects outward, bending fingers up and down asynchronously for moving and twisting objects (Movies S2 and S3, Supporting Information). The grabbing and stretching range of the fingertip is 210 mm, spanning from 35 to 245 mm. The gripper can achieve a twisting angle of [−43°, 68°] when interacting with a ball of 11 mm in diameter. By coordinating these manipulation capabilities, this soft gripper can perform complex tasks even in narrow spaces.

To validate the gripper's performance, we demonstrate two cases of narrow‐space operational tasks, including the grabbing and cross‐obstacle manipulation, and the continuous manipulation of grabbing, placing, and moving objects. As shown in Figure 7c, which depicts the experimental setup, both the task spaces are in front of the baffle, and this soft gripper is positioned behind the baffle. In this platform, the baffle restricts the operation possibility except for the 70 mm slit at the bottom so that the gripper must pass through the slit and execute these tasks through fingertip motions. In the gabbing and cross‐obstacle task shown in Figure 7d, the gripper uses its flat body to pass through the slit, and grabs the cup behind the baffle by fingertip opening and closing. Then, two fingers bend upward synchronously in an arc shape to lift the object, while maintaining an unchanged‐height finger root. Within this pose, the gripper enables transportation for objects in the narrow space to cross the obstacle along the moving path. When reaching the designated position, the object is placed and released steadily through the opposite operation of the gripper's fingers. The whole process of this demonstration is shown in Movie S4 (Supporting Information). Besides, the developed gripper capability for multimodal operations realizes the continuous manipulation of multiple objects within the same experimental setup. As demonstrated in Figure 7e and Movie S5 (Supporting Information), the gripper grabs and lifts a Ping‐Pong ball, moving it to the top of an empty cup to release the ball inside. Then two fingers open and bend down to surround the cup, to further grab and lift these two objects for transportation, while putting down and releasing them as they arrive at the targeted position. The versatility and complexity of these operations in narrow spaces are made possible by the gripper's multimodal capabilities, which stem from the proposed McFI actuator. This comprehensive demonstration exhibits the potential for responding to complex environments and tasks.

Furthermore, the integrated sensing function of the McFI actuator, which estimates its motion was used to endow the gripper with an exteroception function, promoting the gripper to detect the interacted‐object size. Specifically, the gripper utilizes its fingertip motions to interact with objects through inward grabbing and outward stretching. Once the gripper interacts with a certain‐width object, the movements of the gripper would be limited, even with continuous pressurization and depressurization on active cavities. At this time, the McFI actuator with definite displacement can be sensed based on the validated sensing model, while the interacted‐object width represented by two‐fingertip span can be further estimated. For this exteroception, it is necessary to establish the geometric relationship between the McFI actuator's displacement and the distance between two fingertips. Through the kinematic analysis of this gripper, as shown in Figure 7f, the exteroception model of the object's width *D* from the passive‐cavity pressure can be derived combined with Equation (3), as follows:

(11) $$\begin{array}{ccc} D & = & 2\left(l_{3}-l_{2}\cos\left(2\pi -\theta_{1}-\theta_{2}-\left(\frac{h\left(P_{p0}-P_{p}\right)}{l_{1}\varepsilon_{V}\left(P_{p}+P_{AT}\right)}+\frac{h}{l_{1}}\right)\right)\right)-2r,\;\mathrm{Grab}\\ & = & 2\left(l_{3}-l_{2}\cos\left(2\pi -\theta_{1}-\theta_{2}-\left(\frac{h\left(P_{p0}-P_{p}\right)}{l_{1}\varepsilon_{V}\left(P_{p}+P_{AT}\right)}at+\frac{h}{l_{1}}\right)\right)\right)+2r,\;\mathrm{Stretch} \end{array}$$

where {*l*_1_,*l*_2_,*l*_3_,θ_1_,θ_2_,*r*} is geometry parameters of this gripper design and is constant. *r* represents the radius of the fingertip, which uses different sides for interaction during the grabbing and stretching process, deriving the two‐cases Equation (11).

To verify this model, we conducted experiments in which the gripper interacted with various objects with widths from 70 to 170 mm. These experiments were grouped into two similar operation cases of grabbing and stretching, as shown in Figure 7g. During the experimental process, the gripper closes/expands to grab/stretch the object, keeps, and finally releases the object, resulting in the variation of pressure feedback from the passive cavity. We recorded this feedback in real‐time and simultaneously converted it into predicted values based on the derived model (11). The values at the interaction inflection points were used to determine the perceived object's width. The experimental result in Figure 7h showcases that the gripper accurately estimates objects’ width ranging from 70 to 140 mm during inward grabbing, and 110 to 170 mm during outward stretching. Their RMSE are 2.5 and 2.3 mm within inward grab and outward stretch, respectively. These experiments demonstrate the effectiveness and accuracy of the developed gripper's exteroceptive function, enabling real‐time prediction, that is free from the disturbance of unknown interaction force and has an extensive sensing‐width range, which is benefited by the proposed McFI actuator.

## 3 Conclusion and Future Work

This paper presents a McFI approach to achieve the integration of multidimensional motions and simultaneous sensing for soft actuators. Inspired by human muscle bundle, a BIFE is proposed to form the cavities within the actuator chamber and reinforce the desired motions. By analyzing the multicavity influence on the strains of folding deformation, an optimized three‐cavity design is developed for the McFI actuator prototype. Due to the airtightness requirements of the multicavity and complex interweaving structure, FDM 3D printing fabrication was utilized to fabricate the actuators. The McFI actuator with a displacement range of [−27.5, 15] (mm), achieves 3‐DOF movement, including axial translation and bidirectional bending, while simultaneously integrating sensing capability for displacement and force. The kinematic and force models are derived analytically to establish the real‐time mapping relationships from multicavity pressures to the actuator's output displacements and force. With a series of experimental validations, the proposed McFI actuator exhibits simultaneous motions and sensing capabilities, showing accurate sensing performances for displacement, spatial orientation, and axial force, with RMSE of 1.0 mm, 6.5°, and 4.0 N, respectively. Especially, the McFI actuator can synchronously estimate displacement and force during linear motion (RMSE of 1.1 mm and 4.0 N respectively), even with the obstacle placed at a random distance (set at −10 mm from the end of actuator).

Two robotic applications, a soft crawling robot, and a narrow‐maneuverable gripper, are developed and driven by the McFI actuators, demonstrating multimodal operations and integrated perception for their locomotion and manipulations, respectively. The soft crawling robot performs planar movements, including forward and backward motion and bidirectional turns, showing dexterous locomotive ability with a maximum moving and turning stride of 33.6 mm and 92°, respectively. This soft robot has the validated ability to accurately perceive and synchronously reconstruct the crawling path, with the RMSE of 6.8° in spatial‐orientation perception, highlighting its integrated functionality. The narrow‐maneuverable gripper showcases multimodal operations, namely, closing fingers for inward grab, extending fingers for outward stretch, asynchronous bending for the object's lateral translation and twisting, and perceiving the object's width. With such integrated exteroception in large operating space, this gripper is able to estimate the object's width from 70 to 140 mm with RMSE = 2.5 mm during inward grab, and from 110 mm to 170 mm with RMSE = 2.3 mm when outward stretch, demonstrating its potential for intelligent interaction with objects.

These experimental results prove the effectiveness of the proposed McFI approach for constructing compact functional soft actuators and enabling soft robots with dexterous movements and integrated perception. Such McFI actuators have the potential to realize the closed‐loop control and act as a stand‐alone function‐integrated module in soft robotic systems without additional sensors, paving the way to develop highly integrated and intelligent soft robots.

Future work will explore the optimized foldable structure design and the effect of different cavity arrangements for function customization, aiming to achieve a systematic design approach for such soft actuators. The force sensing model will be expanded to estimate spatial force perception. A comprehensive closed‐loop control strategy based on sensing feedback will be investigated to achieve precisely controllable motion and interaction. We will also implement the McFI actuators into various robotic configurations to develop intelligent soft robotic systems for complex application requirements.

## 4 Experimental Section

### Simulation of the McFI Actuator

To explore the multicavity deformation effect within the McFI actuator, the Abaqus 2021 (Dassault Systemes) was used for simulation to reveal its strain distribution during pneumatic actuation visually. These were carried out in Abaqus/Standard modal, and the material of the McFI chamber was set as elastic with Young's Modulus 20 MPa and Poisson's ratio of 0.47. The meshing element was the tetrahedral type with an approximate global size of 1.5.

### Pneumatic Actuation Setup and Strategy

The automatic actuation system mentioned in Figure 4 adopts the pump‐valve actuation method. It enables controllable opening and closing adjustment of the valve by controlling the on‐off of the relay board through MCU (STM 32 F103ZET6, ST Microelectrons Inc.). As for each actuation channel, when the controller sets the desired pressure value *P_d_*, it will determine whether to open the corresponding valve by judging the difference between the current pressure *P_c_* in the channel and the desired value. If the pressure difference meets the relationship of |*P_c_* − *P_d_* | > *P_e_*, valves for positive/negative pressure were opened accordingly and closed otherwise, where *P_e_* = 1.5*kPa* was a self‐defining constant representing the dead‐zone range of pneumatic actuation. Among them, each actuation channel was equipped with two valves, connected to a positive pressure source and negative pressure source, in which the pressure source steadily maintains their relative pressure of 120 kpa of positive one and −90 kpa of negative one, supplied by pumps.

### Experimental Sensors, Signal Acquisition, and Processing

The experimental information mainly includes real‐time information for the actuation input, output performance, and state during the soft actuator and developed robot performing motion and interaction, including pneumatic pressure, displacement, interaction force, motion angle, etc. Thus, different sensors were used to obtain the corresponding raw data, with the pressure sensor (XGZP6857A, ±100 kPa, CFSensor Inc.) for pneumatic pressure of actuators, the laser sensor (HG‐C1100, Panasonic Inc.) for linear displacement, the 1D force sensor (AR‐DN103, 0–100 N, 0.2%, Arizon Inc.), the IMU (MPU6050, InvenSense Inc.) for angle, and the camera (D450, Realsense Inc.) for spatial position of the actuator's endpoint.

Excepted the IMU and camera, the data obtained by these sensors was analog, which was collected by the ADC module board (AD7606, ADI Inc.) and was transmitted to the MCU, while the IMU's data was transmitted through serial communication and the MCU transmits these data to PC through serial port to realize the signal acquisition. Data was processed on MATLAB (MATLAB R2023b, MathWorks, USA) to obtain all analysis results and graphs.

## Conflict of Interest

The authors declare no conflict of interest.

## Supporting information



Supplemental Movie 1

Supplemental Movie 2

Supplemental Movie 3

Supplemental Movie 4

Supplemental Movie 5

## Data Availability

The data that support the findings of this study are available in the supplementary material of this article.
